# Anti‐MDA5 antibody‐positive clinically amyopathic dermatomyositis with diffuse alveolar damage diagnosed by transbronchial lung cryobiopsy: A case report

**DOI:** 10.1002/rcr2.865

**Published:** 2021-10-19

**Authors:** Hiroyuki Ishihara, Kensuke Kataoka, Reoto Takei, Yasuhiko Yamano, Toshiki Yokoyama, Toshiaki Matsuda, Tomoki Kimura, Junya Fukuoka, Takeshi Johkoh, Yasuhiro Kondoh

**Affiliations:** ^1^ Department of Respiratory Medicine and Allergy Tosei General Hospital Seto Japan; ^2^ Department of Laboratory of Pathology Nagasaki University Hospital Nagasaki Japan; ^3^ Department of Radiology Kansai Rosai Hospital Amagasaki Japan

**Keywords:** anti‐melanoma differentiation‐associated gene 5, clinically amyopathic dermatomyositis, cryobiopsy, diffuse alveolar damage, rapidly progressive interstitial lung disease

## Abstract

Diffuse alveolar damage (DAD) is known to be a pathological hallmark of acute respiratory distress syndrome or acute interstitial pneumonia, and to have a poor prognosis. We report a case of anti‐melanoma differentiation‐associated gene 5 (MDA5) antibody‐positive clinically amyopathic dermatomyositis (CADM) with rapidly progressive interstitial lung disease (RP‐ILD), in which DAD was confirmed by transbronchial lung cryobiopsy at an early stage without respiratory failure. Although this patient initially did not show respiratory failure, his respiratory condition gradually worsened despite intensive immunosuppression therapy and he died 3 months later. Therefore, the early pathological findings of DAD did not match the clinical picture, which showed no respiratory failure. However, these findings were consistent with the subsequent course and poor outcome. Histological DAD, even in the absence of respiratory failure, may indicate a subsequent poor prognosis and explain the refractory course of RP‐ILD with anti‐MDA5 antibody‐positive CADM.

## Graphical abstract

We report a case showing diffuse alveolar damage in a patient with rapidly progressive interstitial lung disease (RP‐ILD) with anti‐melanoma differentiation‐associated gene 5 antibody‐positive clinically amyopathic dermatomyositis, diagnosed by transbronchial lung cryobiopsy (TBLC) at an early stage without respiratory failure. Although this patient initially did not show respiratory failure, his respiratory condition gradually worsened despite intensive immunosuppression therapy and he died 3 months later. In patients with RP‐ILD, TBLC has the potential to predict prognosis and treatment response by evaluating the pathological pattern.

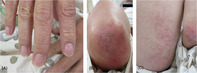

## INTRODUCTION

Anti‐melanoma differentiation‐associated gene 5 (MDA5) antibodies are associated with clinically amyopathic dermatomyositis (CADM), which is characterized by typical cutaneous manifestations of dermatomyositis with little or no muscle abnormalities.^1^ Some anti‐MDA5 antibody‐positive CADM cases are associated with rapidly progressive interstitial lung disease (RP‐ILD)^1^ and poor prognosis. Autopsy findings in these cases sometimes showed a pattern of diffuse alveolar damage (DAD).^2^ We report a case showing DAD in a patient with RP‐ILD with anti‐MDA5 antibody‐positive CADM, diagnosed by transbronchial lung cryobiopsy (TBLC).

## CASE REPORT

**FIGURE 1:**
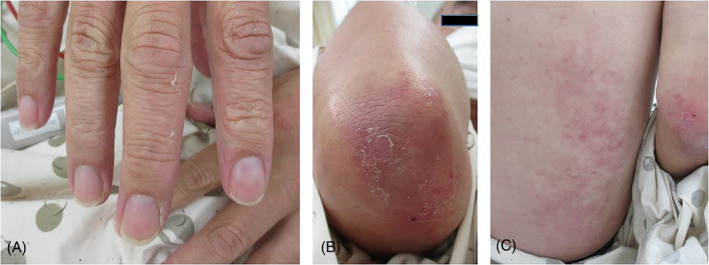
Physical findings. (A) Gottron's sign, (B) rash on the right elbow (Gottron's sign) and (C) rash on the back

**FIGURE 2:**
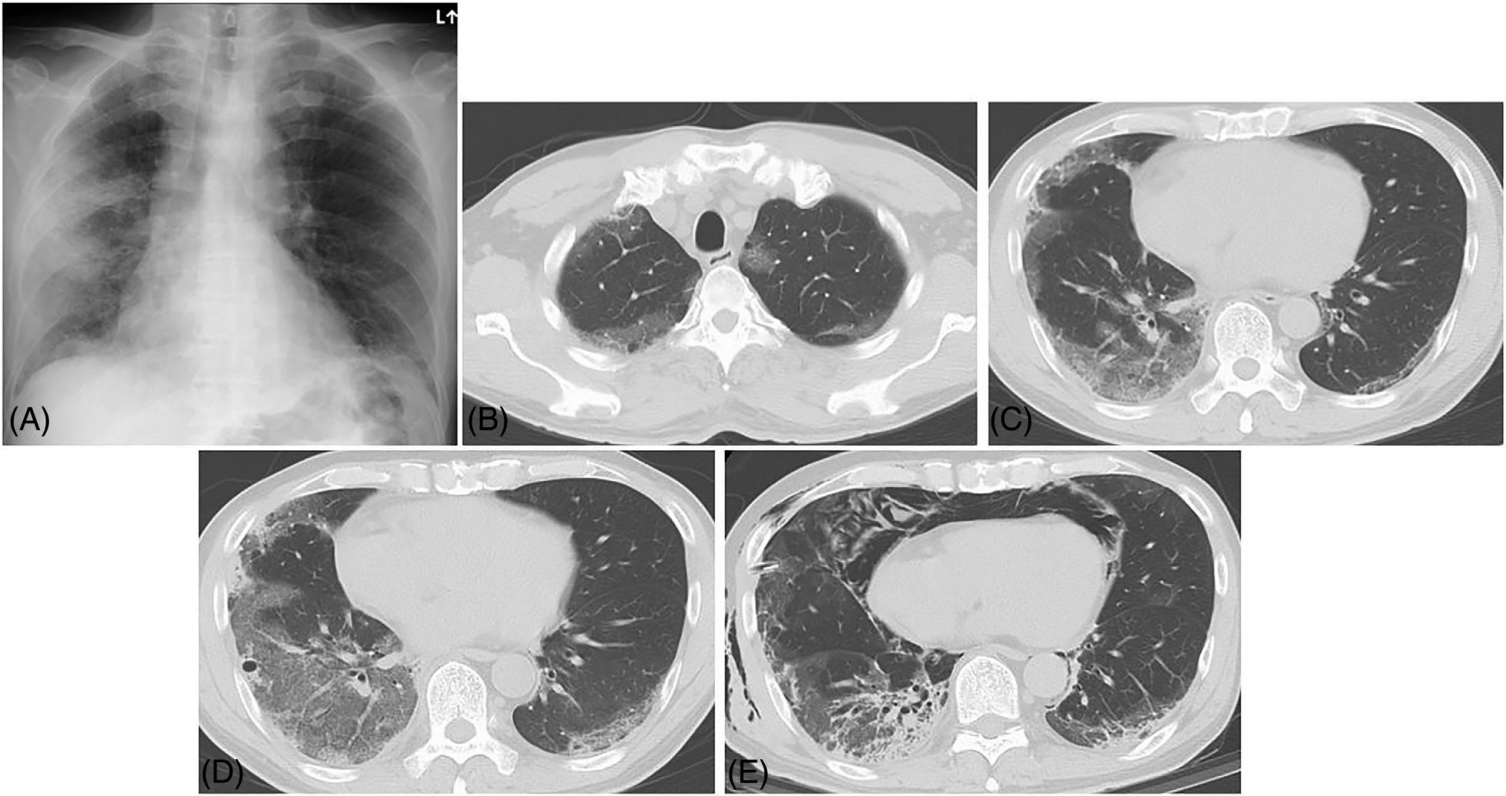
Radiological findings. (A) Chest x‐ray showing bilateral ground‐glass opacity (GGO) which was dominant in the right lung. (B, C) High‐resolution computed tomography (HRCT) on day 1. The area of the perilobular GGO in the upper lobes and lower lobes. (D) HRCT on day 5. The area of the GGO that had spread, and the cavity after cryobiopsy. (E) HRCT on day 23. Radiological fibrosis was remarkable on the lower lobe

A 53‐year‐old man was admitted with a 10‐day history of fever and cough. His respiratory rate was 16 times per minute and his body temperature was 37.5°C. Physical examination revealed fine crackles in the middle to lower lung fields and skin changes. He had violet‐coloured rashes on his knuckles, elbows, knees and back, and showed Gottron's sign (Figure 1). Muscle weakness was not observed. The laboratory findings revealed elevated serum levels of Krebs Von den Lungen‐6 (1375 U/ml), white blood cells (7900/μl), C‐reactive protein (9.13 mg/dl), ferritin (777.8 ng/ml) and aldolase (6.0 IU/L), but creatinine kinase was normal. An arterial blood gas analysis showed normal partial pressure of oxygen (PaO_2_, 65.8 mmHg). Anti‐MDA5 antibody was remarkably elevated (640 index value). Chest x‐ray depicted bilateral ground‐glass shadow (Figure 2A), and a high‐resolution computed tomography (HRCT) scan showed bilateral ground‐glass opacity (GGO) on the peripheral sides (Figure 2B,C). Pulmonary function tests showed that the predicted value of forced volume capacity and the predicted value of diffusing capacity of the lungs for carbon monoxide were 98.4% and 65.0%, respectively. Echocardiography showed no cardiac abnormality.

**FIGURE 3:**
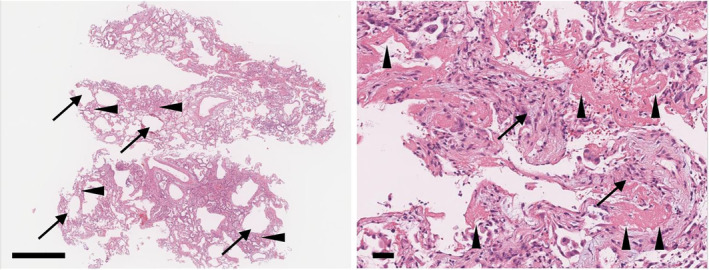
Histopathology of the cryobiopsy. (A) Scanning view of the cryobiopsy. Diffuse abnormality without accentuated localization is seen along with dilatation of alveolar ducts (arrows), which is caused by the collapse of terminal alveolar sacs (arrow heads) (haematoxylin and eosin [H&E], scale bar = 1 mm). (B) High magnification shows numerous hyaline membranes (arrow heads) along with organization of the septa (arrows), which confirm the diagnosis of diffuse alveolar damage (H&E, scale bar = 100 μm)

On further investigation, bronchoalveolar lavage (BAL) in the right B4 and TBLC using a flexible cryoprobe were performed on day 2. The total cell count was 0.83 × 105 cells/ml and lymphocytes (12%), neutrophils (2%) and macrophages (86%) were shown. The culture of the BAL fluid revealed normal flora. Two samples were obtained from the right B8 and B9 by TBLC. Pathologically, the specimens showed diffuse acute abnormality without chronic changes (Figure 3). The tissue had dilatation of alveolar ducts due to the collapse of alveolar sacs, and numerous hyaline membranes along with organization of the septa. A diagnosis of DAD was made. There was no granuloma, intense lymphoplasmacytic infiltration, lymphoid follicles or viral inclusion. Based on the described findings, a final diagnosis of anti‐MDA5 antibody‐positive CADM and RP‐ILD with DAD was made.

The patient was initially treated with combination therapy and immunosuppression using methylprednisolone, tacrolimus, tofacitinib and intravenous cyclophosphamide. During the first month after admission, no hypoxaemia was observed at rest, only during exercise. After the first month, his respiratory condition gradually worsened. Radiological fibrosis was remarkable on the lower lobe (Figure 2E). Pneumothorax occurred on his right lung as a complication of the treatment and chest tube drainage was performed. On day 54, his respiratory state rapidly deteriorated with newly progressive GGO on HRCT. Afterwards, his respiratory condition remained unstable and he died on day 94. The findings from necropsy, which was performed from the right lower lung, showed organizing and organized DAD and fibrosis. Although there was a mild Aspergillus infection in the lung, we concluded that the primary cause of death was not infection but progression of ILD by comprehensive evaluation including necropsy findings.

## DISCUSSION

To our knowledge, this is the first report of a patient with anti‐MDA5 antibody‐positive CADM showing DAD diagnosed by TBLC in the early stage of RP‐ILD without respiratory failure. DAD is known to be a pathological hallmark of acute respiratory distress syndrome (ARDS) and acute interstitial pneumonia (AIP). Almost half of ARDS patients who underwent surgical lung biopsy (SLB) had DAD. The presence of DAD is associated with high mortality both in ARDS and AIP.^3^ Both are characterized by worsening of the respiratory condition in a short period. Our patient did not initially have respiratory failure, which was atypical among patients with DAD in ARDS or AIP. Thus, DAD can be detected in patients with this disease from the early phase without respiratory failure.

Only one case of DAD diagnosed by SLB has been reported in anti‐MDA5 antibody‐positive RP‐ILD patients,^3^ others were reported based on autopsy.^2^ Despite intensive immunosuppression therapy, which has been reported to be efficacious, our patient died. In contrast, a non‐DAD patient with anti‐MDA5 antibody‐positive RP‐ILD who survived has been reported.^4^ It may be supposed that the histological pattern is associated with disease behaviour or prognosis. Our patient also had prognostic factors known to be present in patients with interstitial pneumonia associated with CADM, such as a high level of anti‐MDA5 antibody and serum ferritin and extensive GGO. Further investigations will be needed to confirm the prognostic factors in patients with RP‐ILD with anti‐MDA5 antibody‐positive CADM.

TBLC is reported to be less invasive and to have fewer complications than SLB. In addition, a recent study showed high levels of agreement between TBLC and SLB in both histopathological interpretation and diagnosis.^5^ Therefore, TBLC is increasingly recognized as a potential alternative to SLB for the diagnosis of ILD, especially when SLB may be risky.

In conclusion, this is the first report of a patient with anti‐MDA5 antibody‐positive CADM showing DAD diagnosed by TBLC in the early stage of RP‐ILD without respiratory failure. In RP‐ILD patients, TBLC has the potential to predict prognosis and treatment response by evaluating the pathological pattern.

## CONFLICT OF INTEREST

None declared.

## ETHICS STATEMENT

The authors declare that appropriate written informed consent was obtained for the publication of this case report and accompanying images.
